# Health behaviours and health-related quality of life among women and men with hazardous alcohol use: the Tromsø study (2015-2016)

**DOI:** 10.1186/s12889-026-26515-0

**Published:** 2026-02-16

**Authors:** Mari Tjelta Berge, Torgeir Gilje Lid, Vendela Husberg-Bru

**Affiliations:** 1 Centre for Health Promotion, Stavanger Municipality, Stavanger, Norway; 2 Department of Public Health, Faculty of Health Sciences, University of Stavanger, Stavanger, Norway; 3 Centre for Alcohol and Drug Research, Stavanger University Hospital, Stavanger, Norway

**Keywords:** Hazardous alcohol use, Health behaviours, Health-related quality of life, Population-based study, AUDIT

## Abstract

**Background:**

Hazardous alcohol use poses a great public health burden. This study examined characteristics and group differences in the health behaviours physical activity, fruit and vegetable intake, alcohol intake per gram, and health-related quality of life (HRQOL), among women and men with hazardous alcohol use.

**Methods:**

This cross-sectional study used data from 17 744 women and men (40–99 years) the Tromsø Study (2015–2016). Hazardous alcohol use was defined by the Alcohol Use Disorder Identification Test (AUDIT), with a cut-off score at ≥ 8. Physical activity was defined by Saltin-Grimby Physical Activity Level Scale (SGPALS), fruit and vegetable intake by units, and alcohol intake by grams. HRQOL was defined by EQ‑5D‑5L. Data were analysed by Chi-Square Tests and analysis of covariance.

**Results:**

The hazardous group had higher alcohol intake, slightly lower fruit and vegetable intake, and mixed physical activity patterns. There was a large difference in daily alcohol intake per grams between the groups. There were no significant differences between the groups HRQOL measured by EQ‑5D‑5L index. However, when testing the EQ‑5D‑5L dimensions separately the hazardous group had significantly lower Anxiety/depression, Self-care and Pain/discomfort score. Anxiety and depression were the HRQOL dimension with the largest group difference in the sample.

**Conclusions:**

These results indicate that apart from higher alcohol intake and slightly more anxiety and depression, the health behaviours and HRQOL among individuals with hazardous alcohol were broadly similar to those without hazardous alcohol use.

## Background

Alcohol consumption has increased globally, and is considered a leading risk factor for various diseases, injuries, and mental health problems [1]. Violence, self-harm, and suicide are often associated with high alcohol consumption [2], and the risk of these outcomes increases with higher alcohol intake (dose-response relationship) [3]. Men and younger individuals are disproportionately affected by alcohol-related harm, which may reflect differences in drinking patterns, biological vulnerability, and social context [1]. Between 1990 and 2017 the global adult per-capita alcohol consumption increased from 5.9 to 6.5 L and was forecasted to reach 7.6 L by 2023 [4]. Hazardous alcohol use refers to consuming alcohol beyond a certain amount and pattern, leading to increased risk of harmful events and negative health outcomes [5]. Potential consequences include decreased quality of life [6] and increased disease burden [7]. Early identification of hazardous alcohol use is challenging, particularly in general populations where individuals may not seek help or report symptoms. Examining associated health behaviours and health-related quality of life (HRQOL) may offer indirect indicators of risk, and support the development of effective prevention and treatment strategies.

Health behaviours refers to actions directly impacting health outcomes [8]. Little or no alcohol use [9], sufficient fruit and vegetable intake [10] and being sufficiently physical active [11], are known health protective behaviours. Healthy eating habits and physical activity behaviours are associated with higher odds of quitting hazardous alcohol use [12]. Physical activity combined with other positive health behaviours leads to better health outcomes [13]. While alcohol consumption is associated with inadequate fruit and vegetable intake [14], the link between physical activity and alcohol use remains contradictory. Large population-based studies indicate a positive relationship between physical activity and alcohol intake [15] which may seem counterintuitive given that physical activity is generally considered health-promoting [16]. Early identification of hazardous alcohol use remains a complex and challenging task [17]. Studying health behaviours in relation to hazardous alcohol use may help identify patterns that are not captured by alcohol screening tools alone and provide insight into behavioural profiles that signal elevated risk.

From a nutritional perspective women (31%) are more likely than men (17%) to meet the five-a-day recommendation for fruit and vegetable intake, and odds of compliance with recommended vegetable/fruit/berries intake are positively associated with age, education, and physical-activity levels [18, 19]. This context is relevant when examining whether individuals with hazardous alcohol use deviate from typical intake patterns or reflects broader population trends.

Health behaviours are associated with a multiple of factors, HRQOL being one of them [20]. The EuroQol group defines that HRQOL encompasses five health dimensions: Mobility, self-care, usual activities, pain/discomfort and anxiety/depression [21]. These dimensions describe physical and psychological functioning. Hazardous alcohol use is associated with lower life-satisfaction, reduced well-being and increased psychological distress [22]. Previous research found positive association between HRQOL and light alcohol use, and negative association for higher alcohol use [23].

Men drink alcohol in larger amounts, more frequently, and are at greater risk of alcohol-related harm than women [24]. Drinking frequency increases with age, while hazardous drinking patterns tend to decrease with age [1]. High educational and income are associated with higher drinking frequency, but less risky drinking [1]. Hazardous alcohol use and negative health behaviours are associated with health problems [2], and high body mass index (BMI) with less physical activity [25], and lower fruit and vegetable intake [26]. Frequent high alcohol intake is associated with worse HRQOL and mental distress [27].

Few studies have investigated health behaviours and HRQOL together in the context of hazardous alcohol use among a population-based sample of middle-aged and older adults. Previous studies have predominantly studied either alcohol use and health behaviours [14, 15, 28] or alcohol use and HRQOL [27]. Studies exploring the field have used varying methodologies and study samples, including college students [29], athletes [30], 21–65 years only [15], and are characterized by small sample sizes [30], or clinical samples [23, 31] –not representative to the general population of older adults. By using a large, population-based sample aged 40 and above, this study provides insights into a demographic increasingly relevant for public health planning. Examining health behaviours and HRQOL together allows for a more comprehensive understanding of how hazardous alcohol use may be associated with both lifestyle factors and perceived health status, which is essential for developing targeted interventions. The analytical framework used in this manuscript was first developed in a master’s thesis based on data from the Tromsø Study [32].

The primary aim of this study was to investigate the characteristics and group differences in health behaviours – physical activity, fruit and vegetable intake, and alcohol intake per gram – of participants with a hazardous alcohol use (hazardous group), compared to participants without a hazardous alcohol use (non-hazardous group). The secondary aim was to investigate the characteristics and group differences of HRQOL between these groups.

## Methods

### Design and study sample

This cross-sectional population-based study used data from the seventh survey of the Tromsø study (Tromsø7 2015-16). The Tromsø study is one of Norway`s most comprehensive population-based health surveys, with seven repeated surveys to date (Tromsø1–7 1974–2016) [33], collecting multipurpose health data on chronic and non-communicable diseases and risk factors [34]. Invitations were sent to all 32,591 registered inhabitants in Tromsø municipality aged *≥* 40 years [34] by mail, and included details on completion of online questionnaire before attendance, information brochure, and access to three questionnaires [33, 34]. Physical examination on-site was also conducted [33]. A total of 21,083 women and men aged *≥* 40 years from Tromsø municipality in Northern Norway participated (attendance rate 65%) [34]. The present study uses self-reported data supplemented by BMI. In total, 17 744 of the attenders completed the Alcohol Use Disorder Identification Test (AUDIT) and became the study sample for the present study.

## Measures

### Hazardous alcohol use (Predictor Variable)

Hazardous alcohol use was measured by the Alcohol Use Disorder Identification Test (AUDIT), a scale developed to screen for hazardous and harmful alcohol use [35]. This 10-item scale measures harmful alcohol use and dependence in the domains of quantity and frequency, behaviour, and psychosocial consequences [35]. The AUDIT scale ranges from 0 to 40, where lower scores indicate minimal risk of alcohol-related problems, while higher scores suggest risk of harmful drinking or alcohol dependence [36]. In the present study, the AUDIT was dichotomized with a cut-off at 8, where scores of 7 or below indicate no alcohol-related problems, and scores of 8 or higher indicate hazardous alcohol use, defined as a pattern of drinking that increases the risk of harmful consequences. This does not necessarily imply alcohol dependence. This cut-off has been shown to have high sensitivity and specificity [37] in detecting hazardous alcohol use. The AUDIT is thoroughly validated and demonstrate considerable usefulness when screening for hazardous and harmful alcohol use [38].

### Health behaviours (Outcome variables)

Self-reported leisure time physical activity during the past year was measured by the Saltin-Grimby Physical Activity Level Scale (SGPALS), which is evaluated, and the concurrent validity and predictive validity are shown to be good when compared with aerobic fitness and long-term health outcomes [39]. Response categories were 1: sedentary activities, 2: light physical activity *≥* 4 h/week, 3: regular physical activity/training *≥* 2–3 h/week and 4: regular hard exercise several times/week. As few participants scored in the highest response category, the categories 3 and 4 were collapsed into one category labelled “exercise”, as done in previous studies [40]. SGPALS was used both to describe the sample, and as an outcome variable in the first study aim.

Self-reported intake of fruit or vegetable units per day served as an outcome variable, with a continuous response option. One unit corresponds with 100 g of fruit or vegetables [41]. Additionally, frequencies of fruits/vegetables/berries intake were included for sample descriptives. Response categories: 0–1 pr. Month, 2–3 pr. Month, 1–3 pr. Week, 4–6 pr. Week, 1 or more pr/day.

In addition to the AUDIT, participants self-reported their alcohol intake in units/day (continuous variable). Units were converted into grams. This was used both to describe the sample, and as an outcome variable. In Norway, 12 g of alcohol corresponds to one unit of alcohol, equivalent to 33 cl beer (4.5%)/12 cl wine/4 cl spirit [42].

### HRQOL (Outcome variable)

HRQOL was measured by EQ-5D-5L [21], which encompasses five health state dimensions with five response levels, complemented by a visual analogue scale (VAS) to measure overall status. The five dimensions included: mobility, self-care, usual activities, pain/discomfort, and anxiety/depression. Participants marked their current state of health on a VAS ranging from 0 to 100, (100 = best health imaginable, 0 = worst imaginable). Country-specific value sets are estimated for several countries to derive a health state sum score (EQ-5D-5L index) [21]. As no Norwegian value set exists [43], the English Devlin value set Version 1.2 were applied, which is similar to other western countries’ value sets [44]. The EQ-5D-5L index score ranges from − 0.59 to 1, (1 = best possible health, 0 = like death, negative values=worse than death). The English value set is previously applied on Norwegian data and is considered appropriate for use in this context [43]. The EQ-5D-5L is widely regarded as a concise general health measure [43], validated with good reliability and sensitivity [45, 46].

### Variables used to describe the sample

*Comorbidity*: Comorbidity was defined as a binary variable indicating whether participants reported at least one of the following conditions: cancer, psychological problems, angina, heart attack, hypertension, kidney disease, chronic bronchitis, heart failure, atrial fibrillation, rheumatoid arthritis, arthrosis, or stroke. Response categories: (No/Yes, currently/Previously, not now) were recoded and collapsed into one binary variable (Yes/No) named comorbidity. (comorbidity). Persistent or constantly recurring chronic pain lasting *≥*three months (Yes/No) were also included. Comorbidity was also used as a covariate.

*Mental distress* was measured by the Hopkins Symptoms Checklist-10 (HSCL-10), a shorter version of HSCL-25, derived from HSCL-90 [47]. It comprises 10 items related to anxiety (4 items), depression (5 items), and sleep problems (1 item). Four response categories: (1 = No complaint – 4 = Very much). A mean score was calculated and labelled Mental distress. The score was treated as a continuous variable ranging from 1 to 4. The HSCL-10 demonstrates high internal consistency [48]. HSCL-10 was used to describe the sample and as a covariate in ANCOVA models to adjust for mental distress.

*Medication use*: Variables on use of anti-hypertensive drugs and lipid-lowering drugs were included. 3 response categories: (Never/Now/Previously). These were used as proxies for severe disease, as hyperlipidaemia and hypertension is associated with negative health behaviours [49], and are risk factors for chronic diseases [41].

*Body Mass Index* (BMI) (kg/m^2^) were measured during the physical examination. As self-reported data on diet and physical activity may be under- or overreported [50, 51], BMI was included as a proxy for healthy behaviours. Individuals engaging in leisure time physical activity less likely have obesity (BMI *≥* 30), compared to sedentary individuals [52]. BMI was also used as a covariate. BMI was treated as a continuous variable.

Demography: Age (continuous variable), sex (women/men), marital status, living with spouse/partner (yes/no), and educational level was included for sample description and used as covariates.

### Statistical analysis

Sample descriptives were stratified by sex and by the dichotomised AUDIT variable, indicating hazardous alcohol use, or non-hazardous alcohol use. The primary aim was to compare health behaviours – specifically physical activity level, fruit and vegetable intake, and alcohol intake in grams/day – between individuals with and without hazardous alcohol use. Group differences in physical activity were analysed using a Chi-Square Test. For continuous outcomes (fruit and vegetable intake and alcohol intake in grams/day), Analysis of Covariance (ANCOVA) was used to compare estimated marginal means between groups while adjusting for covariates. For fruit and vegetable intake, the model was adjusted for sex, education, marital status, and BMI. For alcohol intake in grams/day, the model was adjusted for sex, education, marital status, comorbidity, and mental distress.

The secondary aim of HRQOL was to compare health-related quality of life (HRQOL), measured by the EQ-5D-5L index and EQ-5D-5L VAS, between individuals with and without hazardous alcohol use. The EQ-5D-5L index was treated as the dependent variable, and hazardous alcohol use (AUDIT ≥ 8) as the independent variable. Group differences in the five individual EQ-5D-5L dimensions were analysed using Chi-Square Tests. An ANCOVA was conducted to examine differences in the EQ-5D-5L index score between groups. Four models were fitted sequentially: Model 1 included no covariates; Model 2 adjusted for sociodemographic variables (age, sex, education, marital status); Model 3 added comorbidity; and Model 4 included mental distress (measured by HSCL-10). Although anxiety and depression are part of the EQ-5D-5L index, mental distress was measured independently using HSCL-10 and included as a covariate, given its strong association with both hazardous alcohol use and perceived health status. This adjustment aimed to examine whether differences in HRQOL between groups persisted beyond the influence of psychological distress rather than other health dimensions. All analyses were performed using IBM SPSS Statistics version 29. Statistical significance was set at *p* < .05.

## Results

In total, 52.5% of the sample (*N* = 17744) were female, with a mean age of 57.3 years (*SD* = 11,42). Among these, 12.2% (*n* = 2159) had hazardous alcohol use (AUDIT ≥ 8). The group with hazardous alcohol use contained 18.8% (*n* = 1637) of the men, and 5.8% (*n* = 522) of the women. The group with non-hazardous alcohol use contained 81.2% (*n* = 7049) of the men, and 94.2% (*n* = 8536) of the women. Regular physical exercise was 22.4% (*n* = 1864) among women and 35.3% (*n* = 2441) among men in the non-hazardous group, and 19.1% (*n* = 98) among women and 32.5 (*n* = 526) among men in the hazardous group. Units of fruit or vegetables per day was 2.42 (*SD* = 1.55) for women, and 1.83 (*SD =* 1.71) for men in the non-hazardous group, and 2.32 (*SD* = 1.62) for women, and 1.64 (*SD* = 1.47) for men in the hazardous group. Daily alcohol intake was 8.82 g. (*SD* = 9.05) for women, and 12.25 g. (*SD* = 11.67) for men in the non-hazardous group, and 25.31 g. (*SD* = 17.61) for women, and 31.92 g. (*SD* = 24.39) for men in the hazardous group. Regarding HRQOL, the total sum score (EQ-5D-5L index) for the sample was 0.90 (*SD*=0.11). The non-hazardous group had an HRQOL sum score of 0.91 (*SD*=0.10), while the hazardous group scored 0.89 (*SD*=0.12). Mean self-rated health (EQ VAS) was (77.08, *SD* = 16.16) for women, and (77.56, *SD* = 14.89) for men in the non-hazardous group, and (73.88, *SD* = 16.43) for women, and (74.15, *SD* = 15.25) for men in the hazardous group. Additional sample descriptives are provided in Table 1.

**Table 1:** Sample characteristics stratified by AUDIT and sex. The Tromsø study 2015–2016 (*N* = 17744)

	No hazardous alcohol use (*n* = 15585)		Hazardous alcohol use (*n* = 2159)	
	Women (*n* = 7853–8536)^i^	Men (*n* = 6402–7049)^i^	Women (*n* = 489–522)^i^	Men (*n* = 1514–1637)^i^
Age, years (SD)	56.26 (10.69)	57.45 (11.20)	51.43 (8.14)	53.67 (9.29)
Education, %				
Primary school	20.6 (1745)	20.8 (1452)	12.3 (64)	18.7 (304)
Secondary school	25.6 (2164)	30.3 (2112)	27.2 (141)	32.0 (520)
College/university < 4 years	18.2 (1539)	22.0 (1536)	20.8 (108	21.8 (354)
College/university > 4 years	35.6 (3014)	26.8 (1871)	39.7 (206)	27.5 (446)
Live with spouse, %	74.5 (5960)	84.2 (5784)	69.0 (329)	74.8 (1176)
Marital status, %				
Single	22.8 (1950)	23.1 (1629)	33.7 (176)	32.7 (536)
Married/Registered partnership	54.0 (4610)	62.7 (4421)	41.6 (217)	50.6 (829)
Widow/Widower	5.9 (501)	1.9 (135)	2.1 (11)	1.0 (16)
Divorced	15.8 (1352)	11.0 (776)	20.1 (105)	13.6 (222)
Separated	1.4 (123)	1.2 (88)	2.5 (13)	2.1 (34)
Medication, %				
Anti-hypertensive drugs	20.5 (1754)	26.4 (1863)	15.5 (81)	22.2 (363)
Lipid-lowering drugs	11.5 (981)	17.5 (1237)	6.7 (35)	13.1 (214)
Comorbidities ^a,^ %	56.2 (4797)	52.0 (3664)	59.2 (309)	53.6 (877)
Pain last > 3 months, %	42.1 (3309)	29.4 (1880)	41.3 (202)	37.2 (563)
Mental distress (HSCL-10)^b,^ (SD)	1.33 (0.38)	1.20 (0.30)	1.60 (0.55)	1.38 (0.45)
Body Mass Index (BMI)^c,^ (SD)	26.75 (4.84)	27.72 (3.95)	26.86 (4.83)	28.25 (3.94)
Units of fruit or vegetables per day ^d,^ (SD)	2.42 (1.55)	1.83 (1.71)	2.32 (1.62)	1.64 (1.468)
Fruit, vegetables, and berries %				
0–1 times per month	0.2 (17)	0.9 (62)	0.2 (1)	1.0 (17)
2–3 times per month	0.9 (78)	3.3 (234)	1.3 (7)	4.8 (78)
1–3 times per week	8.8 (750)	22.2 (1556)	13.3 (69)	27.1 (441)
4–6 times per week	19.7 (1674)	32.2 (2259)	21.8 (113)	31.7 (516)
Once a day or more	70.3 (5967)	41.3 (2896)	63.4 (329)	35.4 (577)
SGPALS_3_T7 ^e,^ %				
Inactive	12.2 (1014)	13.9 (961)	16.1 (83)	19.4 (314)
Light physical activity	65.5 (5450)	50.9 (3520)	64.8 (333)	48.1 (778)
Exercise	22.4 (1861)	35.3 (2441)	19.1 (98)	32.5 (526)
Alcohol, g/day^f,^ (SD)	8.82 (9.05)	12.25 (11.67)	25.31 (17.61)	31.92 (24.39)
Health-Related Quality of Life (EQ-5D-5L index)^g,^ (SD)	0.89 (0.11)	0.92 (0.09)	0.87 (0.12)	0.89 (0.12)
Self Rated Health (EQ VAS)^h,^ (SD)	77.08 (16.16)	77.56 (14.89)	73.88 (16.43)	74.15 (15.25)

Numbers are means for continues variables (standard deviation) and proportion (number) for categorical variables

^a^ Comorbidities includes having at least one of the following conditions: Cancer, psychological problems, angina, heart attack, hypertension, kidney disease, chronic bronchitis, heart failure, atrial fibrillation, rheumatoid arthritis, arthrosis, or stroke

^b^ Mental distress was mean scored, range 1–4

^c^ Body mass index was calculated as weight per square height (kg/m^2^)

^d^ One unit of fruit or vegetable is defined as 100 g

^e^ Exercise and physical exertion in leisure time was measured by Saltin Grimby Physical Activity Scale (SGPALS). The two highest levels were here combined and referred to as exercise

^f^ One unit of alcohol contains ca. 12 g. alcohol, corresponding to 33 cl beer/12 cl wine/4 cl spirit, according to the Norwegian Directorate of Health

^g^ EQ-5D-5L index: Summarised using the English EQ-5D-5L value set, range minimum − 0,035 – maximum 1,0

^h^ EQ-VAS is a 0-100 scale where participants indicate their overall health

^i^*n* was lowest for Pain lasting > 3 months, and highest for age

### Group differences in health behaviour

A Chi-Square Test for Independence (with Pearson Chi-Square) on AUDIT and SGPALS indicated a significant association between hazardous alcohol consumption and physical activity level, χ^2^(1, *n* = 17379) = 59.32, *p* = < 0.001. The effect size was small (*Cramer`s V*=0.06). A larger proportion of the hazardous group were inactive (18.6% versus 13.0%), with less light physical activity (52.1% versus 58.8%) and slightly more moderate to high levels of physical activity compared to the non-hazardous group (29.3% versus 28.2%). See Table 1.

Analysis of Covariance (ANCOVA) was conducted to examine the mean variances in fruit and vegetable intake between the hazardous and the non-hazardous groups, controlling for sociodemographic variables (sex, education, marital status) and BMI. After adjusting for covariates, the fruit and vegetable intake of the non-hazardous group had an estimated marginal mean (EMM) of 2.13 units (95%CI = 2.11, 2.16) while the EMM for the hazardous group were 1.98 units (95%CI = 1.91, 2.05). The hazardous group had a slightly lower intake of fruit and vegetables when controlling for sociodemographic factors and BMI. F(1, 17274) = 15.23, p = < 0.001. See Table 2.

**Table 2:** ANCOVA of Hazardous alcohol use^a^, and fruit and vegetable intake in units/day. The Tromsø Study 2015-2016 (*N*=17471)

	Model 1				Model 2				Model 3			
	EMM^b^	SE	95%CI	*p*	EMM	SE	95%CI	*p*	EMM	SE	95%CI	*p*
Non-hazardous alcohol use	2.15	.01	2.13,2.18	<.001	2.13	.01	2.11,2.16	<.001	2.13	.01	2.11,2.16	<.001
Hazardous alcohol use	1.81	.04	1.74,1.88	<.001	1.98	.04	1.91,2.05	<.001	1.98	.04	1.91,2.05	<.001

^a^ Hazardous alcohol use was defined by Alcohol Use Disorder Identification Test (AUDIT) score of>8

^b^ EMM = Estimated Marginal Means

Model 1= Predictor (AUDIT), and dependent variable (Fruit and vegetable units/day). *F*(1, 17469)=82.53, *p*=<.001

Model 2= Sociodemographic variables (age, sex, education, and marital status).* F*(1, 17312)=15.72, *p*=<.001

Model 3= BMI.* F*(1, 17274)=15.23, *p*=<.001

To examine the mean variances of alcohol intake in grams/day between the hazardous and the non-hazardous groups an ANCOVA was conducted, controlling for sex, education, marital status, comorbidity and mental distress. The EMM of the non-hazardous group was 10.51 g (95%CI = 10.31, 10.70), while the hazardous group had an EMM of 29.64 g (95%CI = 29.09, 30.19). There was a large difference in alcohol intake in grams between the hazardous and the non-hazardous group when controlling for covariates. F(1, 17463) = 4070.66, p = < 0.001. See Table 3.

**Table 3:** ANCOVA of Hazardous alcohol use^a^, and fruit and alcohol intake in grams/day. The Tromsø Study 2015-2016 (*N*=17744)

		Model 1				Model 2					Model 3			
		**EMM**^b^	**SE**	**95%CI**	**p**	**EMM**		**SE**	**95%CI**	**p**	**EMM**	**SE**	**95%CI**	**p**
Non-hazardous alcohol use		10.37	0.10	10.17, 10.57	< 0.001	10.48		0.10	10.28, 10.674	< 0.001	10.51	0.10	10.31, 10.70	< 0.001
Hazardous alcohol use		30.32	0.27	29.79, 30.86	< 0.001	29.67		0.28	29.13, 30.21	< 0.001	29.64	0.28	29.09, 30.19	< 0.001

^a^ Hazardous alcohol use was defined by Alcohol Use Disorder Identification Test (AUDIT) score of > 8

^b^ EMM = Estimated Marginal Means

Model 1 = Predictor (AUDIT), and dependent variable (Alcohol g/day). *F*(1, 17742) = 4684.38, *p* = < 0.001

Model 2 = Sociodemographic variables (age, sex, education, and marital status). *F*(1, 17570) = 4230.59, *p* = < 0.001

Model 3 = Comorbidity and mental distress (HSCL-10). *F*(1, 17463) = 4070.66, *p* = < 0.001

### Group differences in HRQOL

A Chi-Square Test for Independence (with Pearson Chi-Square) where conducted on each of the five dimensions in EQ-5D-5L to measure the group differences of state of health and hazardous alcohol use, as shown in Table 4. In summary, three of the EQ-5D-5L dimensions were statistically significantly associated with hazardous alcohol use: self-care problems, pain and discomfort, and anxiety and depression. Differences for all dimensions were small, but larger for anxiety and depression than for the rest.

The models 1–3 of the ANCOVA on EQ-5D-5L index initially yielded that the hazardous group exhibited a higher EQ-5D-5L index score compared to the non-hazardous group. However, after adjusting for mental distress, this difference disappeared. See Table 4.

**Table 4:** EQ-5D-5L frequencies and proportions reported by dimension and level, stratified by AUDIT^a^, and tested with Chi-Square Test. The Tromsø Study 2015-2016 (*N*=17323)

Dimension	No hazardous alcohol use, *n* (%)	Hazardous alcohol use, *n* (%)	95% *CI*	*P-* value
Mobility				
No problems	12,994 (83.9)	1774 (82.9)	0.77, 0.79	0.774
Slight problems	1937 (12.5)	281 (13.1)		
Moderate problems	398 (2.6)	61 (2.9)		
Severe problems	136 (0.9)	21 (1.0)		
Unable to walk about	20 (0.1)	2 (0.1)		
Total	15,485 (100)	2139 (100)		
Self-care				
No problems	14,908 (96.5)	2029 (95.2)	0.01, 0.01	0.006
Slight problems	470 (3.0)	87 (4.1)		
Moderate problems	43 (0.3)	8 (0.4)		
Severe problems	16 (0.1)	7 (0.3)		
Unable to wash or dress	9 (0.1)	1 (0.0)		
Total	15,446 (100)	2132 (100)		
Usual activities				
No problems	13,067 (84.6)	1756 (82.6)	0.11, 0.12	0.118
Slight problems	1848 (12.0)	295 (13.9)		
Moderate problems	405 (2.6)	54 (2.5)		
Severe problems	108 (0.7)	18 (0.8)		
Unable to do usual activities	22 (0.1)	4 (0.2)		
Total	15,450 (100)	2127 (100)		
Pain/discomfort				
No pain/discomfort	5278 (34.1)	662 (30.9)	0.02, 0.03	0.025
Slight pain/discomfort	7735 (50.0)	1097 (51.3)		
Moderate pain/discomfort	1942 (12.6)	299 (14.0)		
Severe pain/discomfort	462 (3.0)	76 (3.6)		
Extreme pain/discomfort	43 (0.3)	6 (0.3)		
Total	15,460 (100)	2140 (100)		
Anxiety/depression				
Not anxious/depressed	12,426 (80.4)	1422 (66.5)	< 0.001, < 0.001	< 0.001
Slightly anxious/depressed	2536 (16.4)	514 (24.0)		
Moderately anxious/depressed	407 (2.6)	166 (7.8)		
Severely anxious/depressed	76 (0.5)	33 (1.5)		
Extremely anxious/depressed	10 (0.1)	3 (0.1)		
Total	15,455 (100)	2138 (100)		

^a^ Hazardous alcohol use was defined by Alcohol Use Disorder Identification Test (AUDIT) score of > 8

## Discussion

The main findings from this cross-sectional population-based study, were that the health behaviours of the hazardous alcohol group (AUDIT ≥ 8) involved being either more physically inactive or highly physically active, eating less fruit and vegetables, and drinking higher amounts of alcohol in grams, compared to the non-hazardous group. The group differences were large for alcohol intake, but small for physical activity and fruit and vegetable intake. The same accounts for health-related quality of life (HRQOL), where the hazardous group initially had lower HRQOL compared to the non-hazardous group. However, this difference was reduced when adjusted for mental distress, consistent with anxiety and depression representing the HRQOL dimension with the largest group differences. See Table 5. The association between hazardous alcohol use and HRQOL appears to be primarily linked to elevated levels of anxiety and depression.

**Table 5:** ANCOVA of Hazardous alcohol use^a^, and Health-Related Quality of Life^b^. The Tromsø Study 2015-2016 (*N*=17323)

	**Model 1**				**Model 2**				**Model 3**				**Model 4**			
	**EMM**^c^	**SE**	**95%CI**	***p***	**EMM**	**SE**	**95%CI**	***p***	**EMM**	**SE**	**95%CI**	***p***	**EMM**	**SE**	**95%CI**	***p***
Non-hazardous alcohol use	.91	.00	.91, .91	<.001	.91	.00	.91, .91	<.001	.91	.00	.91, .91	<.001	.91	.00	.91, .91	.713
Hazardous alcohol use	.89	.00	.88, .89	<.001	.88	.00	.88, .89	<.001	.89	.00	.88, .89	<.001	.91	.00	.90, .91	.713

^a^ Hazardous alcohol use was defined by Alcohol Use Disorder Identification Test (AUDIT) score of>8

^b^ Health-Related Quality of Life was measured by EQ-5D-5L index, summarised using EQ-5D-5L value set for England, range minimum -0.035 – maximum 1.000

^c^ EMM = Estimated Marginal Means

Model 1= Predictor (AUDIT), and dependent variable (EQ-5D-5L index). *F*(1, 17321)=70.05, *p*=<001

Model 2= Sociodemographic variables (age, sex, and education).* F*(1, 17159)=128.56, *p*=<.001

Model 3= Comorbidities. *F*(1, 17158)=98.25, *p*=<.001

Model 4= Mental distress (HSCL-10). *F*(1, 17066)=.135, *p*=.713

The current study diverges from previous studies regarding their operationalization of study variables. Therefore, direct comparisons are not possible. In addition, many of the studies have been done on clinical [31] rather than population-based samples. The present study found that the hazardous alcohol group was either more inactive or highly active. Other studies also found divergent results; alcohol consumption was associated with both less [28] and more physical activity [53, 54]. These studies used other alcohol measures and included also younger age groups [28, 53, 54].

The mean fruit and vegetable intake was below public recommendations [41] in both the hazardous and non-hazardous groups, consistent with general population findings globally [14, 55, 56]. However, these studies used other measures of drinking levels [55], wider alcohol use focus [14] or no focus on alcohol at all [56]. It is possible that demographic factors such as a high education level and thereby possibly high levels of health literacy [57] of the study population influenced the results of the previous studies. In our study, education was included as a covariate, and higher educational level was associated with healthier behaviours, such as greater fruit and vegetable intake. This aligns with previous findings that individuals with higher health literacy tend to exercise more [58], and have other beneficial health behaviours [59].

While the AUDIT includes a question on alcohol units consumed on a typical drinking day, it does not provide a direct measure of average alcohol intake in grams per day. Alcohol intake in grams per day was included to isolate the quantitative aspect of consumption, which is not fully captured by the multidimensional AUDIT score.

Studies investigating HRQOL and hazardous alcohol use at the general population level are limited. The present study found significant group differences in HRQOL that became non-significant when adjusting for mental distress. This is important because the study shows that the hazardous group has higher levels of anxiety and depression, as indicated by both HSCL-10 and EQ-5D-5L. This is a relevant finding as it has significant implications for HRQOL, given that anxiety and depression are components of both the EQ-5D-5L index and the broader concept of HRQOL. Elevated mental distress in the hazardous group likely contributed to lower HRQOL, especially in psychological domains, highlighting the need to address mental health to improve quality of life. One study found that higher HRQOL was associated with light alcohol use [23]. Low alcohol consumption compared to non or high consumption can be a marker for good health and an active social life. However, the study is not entirely comparable as it is based on patient samples [23].

Although the present study demonstrates a clear association between hazardous alcohol use and elevated levels of anxiety and depression, the cross-sectional design does not allow for conclusions about causality. It remains unclear whether mental distress is a cause or consequence of hazardous alcohol consumption. On one hand, individuals may use alcohol as a coping mechanism to manage symptoms of anxiety and depression. On the other hand, excessive alcohol use may contribute to the development or worsening of psychological symptoms. The bidirectional nature of this relationship has been suggested in previous research, and future longitudinal studies are needed to disentangle these pathways and better understand the temporal dynamics between hazardous alcohol use and mental distress.

### Strengths and limitations

The large sample size, population-based data, high attendance and the relatively equal proportion of women and men strengthen the validity and generalizability to general populations. As Tromsø7 included only participants aged *≥* 40, results are not generalizable for younger populations. However, with an aging population, it becomes increasingly important to understand the health behaviour of middle-aged and elderly individuals. This age span allows for a focused investigation of hazardous alcohol use among middle-aged and older adults, a group often underrepresented in alcohol research. Another strength is that the study focuses on hazardous alcohol levels, which is associated with health risk, but without necessarily involving dependence or more severe problems [5]. Previous studies have often focused on serious alcohol-related problems [60], individuals in remission for alcohol use disorder [61] or simply alcohol intake [62], and do not include non-hazardous drinkers. This limits the ability to compare across the full spectrum of alcohol use behaviours, which our population-based sample allows. Overall, the measurements used in our study are validated, frequently used, and internationally recognized [38, 39, 45, 46, 48, 63].

Of the 21,083 participants in Tromsø7, 17,744 completed AUDIT, which gives lower statistical power than if all participants had completed AUDIT. However, the AUDIT response rate of 84.16% is still considered substantial. Furthermore, selection bias can occur in population-based data [64]. The Tromsø study has not investigated dropouts, but previous similar population-based studies have found that nonattenders had lower socioeconomic status and higher mortality than participants [64]. Few participants in Tromsø7 scored within the higher regions of AUDIT, which may be due to high socioeconomic status among the participants.

Physical activity levels, fruit and vegetable intake, and alcohol intake were examined with self-reported data. A complete dietary chart would have provided a fuller diet view. This was available, but with fewer respondents. Nutrition research is challenging [65] and full dietary registration is resource-intensive [51]. Measurement errors in self-reported dietary patterns are well documented [66], as participants may not remember their food intake correctly [51]. Social desirability bias may result in systematic overreporting of healthy foods and underreporting of fatty and sugary foods [19]. Dietary questions were therefore limited to fruit and vegetable intake as a proxy for a healthy diet. To reduce the impact of any incorrect reporting, we adjusted for BMI. The same accounts for physical activity levels, were single-item self-report measures tend to underestimate sedentary time when compared to device measured activity [50].

Moreover, alcohol use was measured in two ways by self- reported intake in grams (outcome variable) and with AUDIT (predictor variable). A Phosphatidyl ethanol-test (PEth-test) could have served as an alcohol intake biomarker, providing information on the level of alcohol intake the past two to four weeks [67]. This could have adjusted for possible inconsistency in self-reported consumption [68]. However, the PEth-test does not address the behavioural aspect that AUDIT does, and AUDIT is set in 12-month time frame and thus suitable for capturing habits with a larger time scope. PEth-tests were available in the Tromsø7 dataset, but with smaller sample sizes than AUDIT and the alcohol in grams variable. The WHO-recommended cut-off of ≥ 8 points for hazardous drinking for both men and women were applied to maintain consistency with WHO guidelines [35] and comparability with previous research. Future studies may consider gender-specific cut-offs to further refine risk estimates.

Longitudinal data could have given indications on causality, but this was not an aim for this study. As anxiety and depression turned out to be the HRQOL dimension with the greatest group difference, it may be relevant to investigate causality between mental distress and hazardous alcohol use in future research.

## Conclusions

Respondents with hazardous alcohol use had a higher alcohol intake, a lower fruit and vegetable intake, and were either more inactive or highly active than the non-hazardous group. The hazardous group had lower HRQOL compared to the non-hazardous group. This was due to the prevalence of anxiety and depression, self-care problems and pain/discomfort being significantly higher among the hazardous group. The significant group differences in HRQOL became non-significant when adjusting for mental distress. This was consistent with anxiety and depression being the EQ-5D-5L dimension with the greatest group difference. However, this supports that there is a clear group difference in anxiety- and depression levels. It is not possible to distinguish between health-related quality of life and psychological symptoms, as anxiety and depression are critical components in both measures. Results indicate that health behaviours and HRQOL among individuals with hazardous alcohol use are not very different from individuals with non-hazardous alcohol use. There are significant differences between the groups, but the differences are quite small, except for alcohol intake differences, which are large. The present study provides insights useful in designing better healthcare services and health policies for the *≥* 40 age group.

## Data Availability

The Tromsø Study data are protected and are not publicly available due to data privacy laws. Access to data files for statistical analyses may be given based on application to the Tromsø Study.

## Abbreviations

AUDIT: Alcohol Use Disorder Identification Test
HRQOL: Health-related quality of life
BMI: Body Mass Index
HSCL-10: Hopkins Symptom Checklist 10
SGPALS: Saltin-Grimby Physical Activity Scale
ANCOVA: Analysis of covariance
EQ-5D-5L: EuroQol 5-Dimension 5-Level questionnaire
